# Robots Like Me: Challenges and Ethical Issues in Aged Care

**DOI:** 10.3389/fpsyg.2018.00432

**Published:** 2018-04-03

**Authors:** Ipke Wachsmuth

**Affiliations:** Center of Excellence Cognitive Interaction Technology, Bielefeld University, Bielefeld, Germany

**Keywords:** care robots, artificial agents, companions, consciousness, emotions, ethics

## Introduction

Robots have become a big issue in the twenty-first century, not least in elderly assistance. There are hopes that robots will make aged-care jobs less demanding, for example, they could help senior citizens maintain a longer independent life in their own home, assist caregivers in the nursing home, or provide company to the lonely. However, there are different opinions about the use of robots in our society.

In 2012, a survey was conducted in 27 EU countries to examine the public's attitudes toward robots (Special Eurobarometer 382).^1^ More than 26.000 European citizens responded about the areas where they believe robots should be used as a priority or banned. The survey indicated that, in general, less than one-fourth (23%) of Europeans have a negative opinion of robots. But when asked about the areas in which robots should be banned, more than half (60%) of the respondents stated that the use of robots should be banned in the care of children, elderly, and the disabled. Given these figures, little acceptance can be expected currently for robots in aged care.

Nevertheless, the demographic change in developed countries places ever increasing challenges on the care and support of the elderly. Due to a higher life expectancy and declining birth rates, the proportion of older people compared to the younger ones increases and with it the number of people in need of care. For instance in Germany, 30 years ahead, predictions are that the number of 80 year old citizens will be larger than the next generation of 50 year olds that could support them.^2^ One possible solution considered to meet these challenges is that the use of robots in aged care could help to fill the gap.

## Robots as substitute for human care?

More than three decades ago, Feigenbaum and McCorduck (1983) put forward their vision of a geriatric robot: “It doesn't just bathe you and feed you and wheel you out into the sun when you crave fresh air and a change of scene, though of course it does all those things. The very best thing about the geriatric robot is that it listens. ‘Tell me again,’ it says, ‘about how wonderful/dreadful your children are to you. Tell me again that fascinating tale of the coup of'63. Tell me again…’ And it means it.” (Feigenbaum and McCorduck, 1983, p. 93).

When I read this back then, I was appalled. Would it be ethical to fob off old persons with a robot? And would such a robot ever become reality? Thirty years later, there are robots developed for the daily care activities of persons, like lifting, bathing or feeding, including ethical reflection from the care ethics tradition (Van Wynsberghe, 2013). There are also robots or artificial agents that appear able to engage in conversation with people and keep track of personal information (Mattar and Wachsmuth, 2014; Ng et al., 2017) or that can simulate and trigger empathy in their interactions with humans (Paiva et al., 2017).

Some years ago, I ran an ethics journal club^3^, one issue being how needy old people could be supported by robots. In one session, we discussed two papers, one by Sparrow and Sparrow (2006), who conclude that the use of robots to provide emotional care and companionship to lonely older people would be unethical because of the deceit involved: Making people believe that a robot is something with which they could have a relationship would delude them about the robot's capacities and thus deny their human dignity. In the other paper, by Sharkey and Sharkey (2011), the authors discuss whether the responsiveness of interactive robots to social cues may give the illusion of sentience and thus be deceptive. Yet they suggest that this should not mean to conclude that all such attempts are unethical. They discuss evidence from a variety of studies that elderly people's well-being may benefit from interacting with a robot—even when observers of an old person imagining to have a relationship with a robot might view it as depriving them of dignity.

“Couldn't this be accepted,” one of us said, “when the old person feels good with it?” Then, a dispute began between two students. The first one said, very aroused: “An ever so human-like robot cannot give true caring.^4^ That is deceptive and unethical.” The other student replied: “Would that really be so bad? We humans like to be deceived, for instance in an exciting movie.” Again, the first one: “But it is something else if one allows oneself to be deceived or if one deceives someone else!”—“And what if there were one day robots like me?,” I finally asked. Robots *like me*, that would be: sentient beings; intelligent, autonomous, able to communicate; self-aware and empathetic; acting consciously and intentionally.

In Wachsmuth (2008) I examined the question of how to configure an artificial agent so as to enable the agent to adopt a first-person perspective and develop some kind of consciousness and self-identity. My example was “Max,” a humanoid agent embodied in virtual reality that as such can have at most *simulated* experiences, intentions, emotions, etc. Yet as long an artificial agent does not possess a neurophysiological basis required for qualitative experience, I argued, it could not have consciousness of the phenomenal quality of experience like a human being: For instance, its emotional states can not be subjectively experienced by the agent, whereas a mechanism of (simulated) appraisal analogous to feelings could technically be achieved.

Now, building a “robot like me” would encompass the attempt of not only simulating, but actually creating conscious experience in an artificial system. Metzinger (2013) makes a strong case against bringing a self-conscious robot into existence by pointing out that such attempts are likely to raise ethical problems because they would very probably lead to conscious suffering. Even though the phenomenal qualities of (future) advanced robotic systems may differ from those human beings have, he argues, they might for instance be able to feel sensory pain as *their own* pain if have a first-person perspective. Thus he pleads, *cf*. his principle of “negative synthetic phenomenology,” that “We should not deliberately create or even risk the emergence of conscious suffering in artificial or postbiotic^5^ agents, unless we have good reasons to do so.” (Metzinger, 2013, p. 265). One could argue that suffering is part of the human condition and that “robots like me” (able to consciously suffer) may be more empathetic caregivers responding to the emotional needs of a person than a being without conscious experience. But as such robots do not exist as of now (while the care crisis is real), this point is not relevant for my argument in what follows.

## Must caring be “true” to promote the well-being of elderly?

Objectives of aged care include to promote the well-being (health, happiness) of the elderly. True caring^6^ would involve, at least, conscious experience. In this section I want to argue that it may be sufficient for robots to take part in caring when they behave *as if* they care, i.e., give the illusion that they respond to the feelings and the suffering of their care recipients. By “robots,” here, I refer to a variety of robotic systems specifically designed to assist, or provide companionship to, elderly people (care robots, companions), including autonomous robots that interact and communicate with humans (so-called social robots). If we acknowledge that robots for the time being have no conscious experience resp. (Metzinger's case) *should not* have it, a robot cannot be a “true” caregiver.

Now, must caring be true to promote a care recipient's well-being? This is not always the case in human caring either. Certainly, doctors and nurses alike, as human beings having true experience and emotions, are able to give true care. But they also need to be able to protect themselves from emotional distress in their professional action. In fact, doctors may learn to remain emotionally detached from the suffering of the patient and still engage with their patients' situation (Kerasidou and Horn, 2016), or nurses need to be able to regulate their emotions to preserve a professional distance toward patients (Cecil and Glass, 2015).

Emotional engagement in care is a complex issue. Human caregivers can manifest a variable range of emotional involvement in response to a need or difficulty of the elderly. For a robot (based on current technology) this still seems very difficult to do. Nevertheless, a robot that behaves *as if* it cares^7^ may, in my view, be somewhat compared to a doctor or nurse that refrain from emotional involvement as part of their professional action. It would be unclear (not least for a person suffering from dementia) whether a robot truly experiences or just simulates emotions. Yet the same seems to be the case in our encounters with human beings. Their emotions (or conscious states, or intentions) are not directly observable to us but need to be inferred; we can only judge from what we experience in interaction with others.

Is it ethical, then, to use robots in elder care? The ethical issues discussed above are deception and dignity. From an empirical perspective, these concerns might be mitigated, when there is evidence that those concerned or potentially concerned do not really seem affected by them. In fact some of us are likely to accept, or even *prefer*, a care robot's assistance, when our independence is at risk. Here are some sentiments I heard myself: “I would, in old age, rather have a robot help me to get dressed than show myself naked in front of a nurse.” Or (a 75 year old): “I might prefer a feeding robot over a nurse since I would be in control when the spoon comes and not depend on the caregiver.” Or: “I would not be a burden on a robot.” This is also a matter of dignity. In her last year my old aunt—she was 92 years old—got bedridden in the nursing home and had to wear diapers. On the phone she said to me: “Now they took my dignity, I have to wait to get my diapers changed.” This won't be a solitary case—on the contrary, it concerns the suffering of many people. Would then a care robot take away dignity from the aged person or, on the contrary, help to maintain her dignity and independence?

While such empirical evidence remains to be further substantiated, we also have to see that the currently young ones who grew up with modern technology would probably be more likely to accept robots when old than the current generation of old ones. If only a portion (say, half) of the needy would be happy with a robot, the elderly care crisis might be considerably mitigated. Finally, when we speak about using robots in elder care this should not mean to say that human care would be fully substituted by robots. Care robots would normally be employed under the supervision of caregivers. It goes without saying that most of us would enjoy the company of a living being in old age. But when human care is sparse, robots could assist the needy, or serve as companions in the many hours of loneliness. A geriatric nurse told me: “Care robots don't substitute for the human being—they help when no one else is there to help.”

## Conclusion

In this paper I addressed the issue of whether robots could substitute for human care, given the challenges in aged care induced by the demographic change. Looking at the question whether caring must be true at all to promote a care recipient's well-being, I gave some indication that this is not always the case in human caring either. I argued that ethical issues like deception and dignity might be mitigated by evidence that those concerned or potentially concerned do not really seem affected by them. In conclusion, it is my opinion that, while robots don't appear to be able to give true caring in the foreseeable future, it may be sufficient for robots to take part in caring when they behave *as if* they care, that is, give the illusion that they respond to the feelings and the suffering of their care recipients.

## Author contributions

The author confirms being the sole contributor of this work and approved it for publication.

### Conflict of interest statement

The author declares that the research was conducted in the absence of any commercial or financial relationships that could be construed as a potential conflict of interest.

## Footnotes

^1^https://data.europa.eu/euodp/data/dataset/S1044_77_1_EBS382

^2^https://www.destatis.de/bevoelkerungspyramide/

^3^https://www.techfak.uni-bielefeld.de/ags/wbski/JC_Ethik_SS14.pdf

^4^in German: echte Zuwendung

^5^By “postbiotic,” Metzinger refers to cases of self-conscious systems which may be neither exclusively biological nor exclusively artificial, yet artificial subjects of experience (somewhat like what I mean by saying “robots like me”).

^6^The term “true caring” is used informally here to emphasize that human caring includes being able to understand and respond to the feelings and suffering of others, which I believe cannot go without consciousness.

^7^“cares” in the sense of responding to care recipients' emotional needs, whether in providing companionship or in care activities like feeding.

## References

[B1] Cecil P. Glass N. (2015). An exploration of emotional protection and regulation in nurse-patient interactions: the role of the professional face and the emotional mirror. Collegian 22, 377–385. 10.1016/j.colegn.2014.06.002 26775524

[B2] Feigenbaum E. A. McCorduck P. (1983). The fifth Generation: Artificial Intelligence and Japan's Computer Challenge to the World. Boston, MA: Addison-Wesley Longman.

[B3] Kerasidou A. Horn R. (2016). Making space for empathy: supporting doctors in the emotional labour of clinical care. BMC Med. Ethics 17:8. 10.1186/s12910-016-0091-7 26818248 PMC4728886

[B4] Mattar N. Wachsmuth I. (2014). Let's get personal: Assessing the impact of personal information in human-agent conversations, in Human-Computer Interaction. Advanced Interaction Modalities and Techniques, ed Kurosu M. (Berlin: Springer), 450–461.

[B5] Metzinger T. (2013). Two principles for robot ethics, in Robotik und Gesetzgebung, eds Hilgendorf E. Günther J. P. (Baden-Baden: Nomos), 263–302.

[B6] Ng H. G. Anton P. Brügger M. Churamani N. Fließwasser E. Hummel T. (2017). Hey robot, why don't you talk to me?, in Proc. of the IEEE International Symposium on Robot and Human Interactive Communication (RO-MAN) (Lisbon), 728–731.

[B7] Paiva A. Leite I. Boukricha H. Wachsmuth I. (2017). Empathy in virtual agents and robots: a survey. ACM Trans. Interact. Intellig. Syst. 7:3 10.1145/2912150

[B8] Sharkey A. Sharkey N. (2011). Children, the elderly, and interactive robots. IEEE Robot. Automation Mag. 18, 32–38. 10.1109/MRA.2010.940151

[B9] Sparrow R. Sparrow L. (2006). In the hands of machines? The future of aged care. Minds Mach. 16, 141–161. 10.1007/s11023-006-9030-6

[B10] Van Wynsberghe A. (2013). Designing robots for care: care centered value-sensitive design. Sci. Eng. Ethics 19, 407–433. 10.1007/s11948-011-9343-6 22212357 PMC3662860

[B11] Wachsmuth I. (2008). 'I, Max' - Communicating with an artificial agent, in Modeling Communication with Robots and Virtual Humans, eds Wachsmuth I. Knoblich G. (Berlin: Springer), 279–295.

